# ProtPlat: an efficient pre-training platform for protein classification based on FastText

**DOI:** 10.1186/s12859-022-04604-2

**Published:** 2022-02-11

**Authors:** Yuan Jin, Yang Yang

**Affiliations:** Department of Computer Science and Engineering, Shanghai Jiao Tong University, and Key Laboratory of Shanghai Education Commission for Intelligent Interaction and Cognitive Engineering, Shanghai, 200240 China

**Keywords:** Protein sequence classification, ProtPlat, Pre-training, Web server

## Abstract

**Background:**

For the past decades, benefitting from the rapid growth of protein sequence data in public databases, a lot of machine learning methods have been developed to predict physicochemical properties or functions of proteins using amino acid sequence features. However, the prediction performance often suffers from the lack of labeled data. In recent years, pre-training methods have been widely studied to address the small-sample issue in computer vision and natural language processing fields, while specific pre-training techniques for protein sequences are few.

**Results:**

In this paper, we propose a pre-training platform for representing protein sequences, called ProtPlat, which uses the Pfam database to train a three-layer neural network, and then uses specific training data from downstream tasks to fine-tune the model. ProtPlat can learn good representations for amino acids, and at the same time achieve efficient classification. We conduct experiments on three protein classification tasks, including the identification of type III secreted effectors, the prediction of subcellular localization, and the recognition of signal peptides. The experimental results show that the pre-training can enhance model performance effectively and ProtPlat is competitive to the state-of-the-art predictors, especially for small datasets. We implement the ProtPlat platform as a web service (https://compbio.sjtu.edu.cn/protplat) that is accessible to the public.

**Conclusions:**

To enhance the feature representation of protein amino acid sequences and improve the performance of sequence-based classification tasks, we develop ProtPlat, a general platform for the pre-training of protein sequences, which is featured by a large-scale supervised training based on Pfam database and an efficient learning model, FastText. The experimental results of three downstream classification tasks demonstrate the efficacy of ProtPlat.

**Supplementary Information:**

The online version contains supplementary material available at 10.1186/s12859-022-04604-2.

## Background

For the past few decades, there has been an explosive growth of protein sequences in public databases [1, 2]. However, the progress of protein function analysis is relatively slow, due to the costly and time-consuming biological experiments. To accelerate the studies of protein function, researchers have developed a variety of machine learning methods based on the known data in large databases [3, 4]. They have achieved good results in function-related prediction tasks based on protein sequences, such as protein subcellular localization [22–24], protein structural characteristics prediction [28, 29], and protein–protein interaction prediction [30, 31]. Especially, with the rise of deep neural networks, traditional feature extraction methods have been largely replaced by sequence encoding schemes, like the pre-trained word embeddings techniques [5, 6], which produce dense continuous vectors and obtain much better performance than discrete features [7–9].

Protein sequence classification has widely utilized the technologies of text categorization from natural language processing [41]. Benefitting from deep learning models and word embedding methods, text categorization has achieved great progress, which also brings opportunities for improving the performance of protein classification tasks. However, there are inherent difficulties to adapt the word embedding techniques from natural language processing to protein sequence representation. For one thing, there are no defined words in amino acid sequences, while the pre-training of embedding vectors mostly relies on language modeling, e.g., next word prediction. For another thing, protein sequences have a much smaller alphabet but are quite longer than natural language sentences, which brings new challenges to learning models.

To improve the learning performance of machine learning methods, pre-training is a very effective strategy. Pre-training was first proposed in the computer vision field and achieved good results. In recent years, it has been widely used in various tasks of natural language processing. The pre-trained models often have fast convergence speed and good generalization performance, especially for the tasks with limited training data. The existing pre-training models are mainly unsupervised models, like ELMo [10] and BERT [11], which are computation intensive. For instance, SeqVec [26] introduces the language model ELMo to represent amino acid sequences as embedding vectors to obtain the biophysical properties of proteins; ProtTrans [27] uses various transformer models taken from natural language processing to provide the pre-trained model for amino acid sequences. Both the two methods have a high demand for computation resources. Alternatively, as the protein sequence-based classification tasks share some common sequence features, the pre-training can leverage a large-scale labeled dataset and transfer the knowledge to other small-data problems [35, 36].

Based on this idea, we propose a supervised pre-training platform, ProtPlat. We perform a large-scale pre-training task, protein family classification, to automatically extract the effective information from the protein sequences. Benefiting from the large-scale Pfam database [2] and the FastText library [12], ProtPlat has sufficient data for pre-training word embeddings and a highly efficient classification procedure. Furthermore, the pre-trained model can be applied to various protein classification tasks. We implement a web service, which allows users to upload their training and test data. The training data is used to fine-tune the pre-trained model, and the prediction results on the test data are provided on the website. We evaluate the performance of the platform on three downstream protein classification tasks with different data scales, namely the identification of type III secreted effectors, the prediction of protein subcellular localization, and the recognition of signal peptides. The experimental results show that the platform not only has a high response speed but also improves the accuracy of all these tasks. It can be used as a general platform to improve the task of protein sequence classification.

## Materials

As a large-scale corpus is required for pre-training, we use the Pfam database [2], which is a comprehensive collection of protein families, including over 34 million protein sequences. The family label is the training target. As there are too many labels (17,929 labels in version 32.0, 2019), to avoid issues caused by an extremely imbalanced data distribution (as shown in Table 1), we remove the families whose samples are less than 500, resulting in 7249 protein families with 32,853,084 sequences in our corpus.

**Table 1:** Numbers of protein families with different numbers of sequences in Pfam

# protein sequence	# protein families
< 100	5474
< 200	7433
< 300	8775
< 500	10,523
≥ 500	7249

To assess the performance of the pre-training platform, we experiment with three downstream classification tasks, as described in the following.

*Task I: Identification of type III secreted effectors* The type III secretion system (TTSS) is related to the secretion of virulence factors of many Gram-negative pathogens. The effector proteins of the type III secretion system (T3SEs) are directly secreted from bacterial cells into host cells, and then play roles in disease progression and immune response suppression. Identifying the type III secreted effectors can help reveal the mechanism of TTSS. However, the prediction of T3SEs is a particularly challenging job due to the lack of conserved motif or secretion signal, and the existing methods mainly utilize statistical characteristics of amino acid sequences. Here, we adopt the same dataset as WEDeepT3 [13], including 525 training samples (241 effectors and 284 non-effectors) and 138 test samples. The sequence identity is below 40%. Data statistics are shown in Table 2.

**Table 2:** Dataset of type III secreted effectors

Dataset	T3SE	non-T3SE	Total
Train #	241	284	525
Test #	46	92	138

*Task II: Prediction of subcellular localization* The location of a protein in a cell is closely related to its function. Only in a suitable subcellular location can a protein perform its function correctly. Computational prediction of protein localization in cells has been a hot topic in the field of bioinformatics. Most of the existing tools are based on protein sequences and machine learning methods [23–25, 42, 43]. We use a classic benchmark set, BaCeILo [14], including proteins from animals, fungi, and plants, located at four subcellular compartments, i.e., nucleus, cytoplasm, mitochondrion, and secretory pathway. Data statistics are shown in Table 3. Besides, we also use the latest dataset that is used in DeepLoc [4], including 13,858 protein sequences located at 10 subcellular compartments, i.e., nucleus, cytoplasm, extracellular, mitochondrion, membrane, endoplasmic, plastid, Golgi, lysosome, and peroxisome. The data statistics are shown in Additional file 1: Table S1.

**Table 3:** Datasets of protein subcellular localization^*^

Dataset	cy	mi	nu	Sp	Total
Animals_train	302	153	803	632	1890
Animals_test	137	35	363	172	707
Fungi_train	181	177	589	72	1019
Fungi_test	30	11	122	16	179
Plants_train	52	57	60	35	204
Plants_test	6	10	61	6	83

*cy denotes cytoplasm, mi denotes mitochondrion, nu denotes nucleus, and sp denotes secretory pathway

*Task III: Recognition of signal peptides* Signal peptides are usually located at the N-terminals of protein sequences and generally 5–30 amino acids in length. The main function of signal peptides is to promote the secretion of proteins outside the cell or localize them to certain organelles, so the identification of signal peptides can provide clues for revealing protein functions. We consider two types of signal peptides, i.e., Sec substrates cleaved by SPase I (Sec/SPI) and others using the SignalP 5.0 dataset [15]. The proteins are from Eukarya, Archaea, Gram-positive bacteria, and Gram-negative bacteria. Data statistics are shown in Table 4.

**Table 4:** Datasets of signal peptides

Dataset	Sec/SPI	Others	Total
Archaea_train	10	45	55
Archaea_test	50	132	182
Eukaryotes_train	2404	7409	9813
Eukaryotes_test	210	7247	7457
Gram-negative_train	419	1126	1545
Gram-negative_test	90	693	783
Gram-positive_train	164	370	534
Gram-positive_test	25	364	389

## Methods

### Data preparation

#### Protein sequence segmentation

For text categorization tasks, word features are widely used in the classifiers. Similarly, features are extracted from short peptides (i.e., *k*-mers) for protein sequence classification. The quality of word segmentation may have a substantial impact on the accuracy of protein sequence classification. Therefore, the preprocessing step mainly focuses on the segmentation of protein sequences into *k*-mers. As there is neither a dictionary nor word boundaries in biological sequences, it is difficult to segment out *k*-mers with specific semantic meaning. Instead, the protein sequences are often simply segmented into fragments of fixed length. Two segmentation methods are described in the following.

*Non-overlapping segmentation* In the field of bioinformatics, biological sequences are often segmented into fixed-length *k*-mers for feature extraction [7, 33, 34, 39]. Normally, *k* is less than or equal to 5 and cannot be too large. The reason is that, as *k* increases, the *k*-mer space will increase exponentially, which leads to extremely high dimensionality and difficulties for the classification methods [13, 32–34].

The advantage of the *k*-mer segmentation method is that it is simple and convenient. Each *k*-mer contains not only the information of single amino acids but also the information of their surrounding context. However, the shortcoming of the *k-*mers segmentation method is also obvious, i.e., it only considers the *k-*mers with fixed length. To alleviate this limitation, here we consider the word space including all words whose length is less than or equal to *k*. Take the protein sequence “MASPAAERKS” as an example, when *k* is set to 3, the set of words of this sequence is {M, A, S, P, E, R, K, MA, SP, AA, ER, KS, MAS, PAA, ERK}.

*Overlapping segmentation* Using non-overlapping segmentation, a little shift on the starting site of segmentation may lead to very different segmented words. To avoid such uncertainty, the overlapping segmentation method has also been widely used. This method adopts a sliding window to segment out *k*-mers with a stride of 1. In this way, all the substrings of length *k* in the sequences are considered, which has a larger feature space than non-overlapping segmentation and may lead to redundant information. For the protein sequence "MASPAAERKS", when *k* is still set to 3, the word set becomes {M, A, S, P, E, R, K, MA, AS, SP, PA, AA, AE, ER, RK, KS, MAS, ASP, SPA, PAA, AAE, AER, ERK, RKS}. The overlapping *k*-mer segmentation method can preserve more sequence information than non-overlapping segmentation.

To fully utilize the sequence information, we adopt the overlapping segmentation in ProtPlat. We compare the model performance of using these two segmentation methods. Results are discussed in Sect. 4.4.1.

#### Processing of the Pfam database

**Fig. 1:**
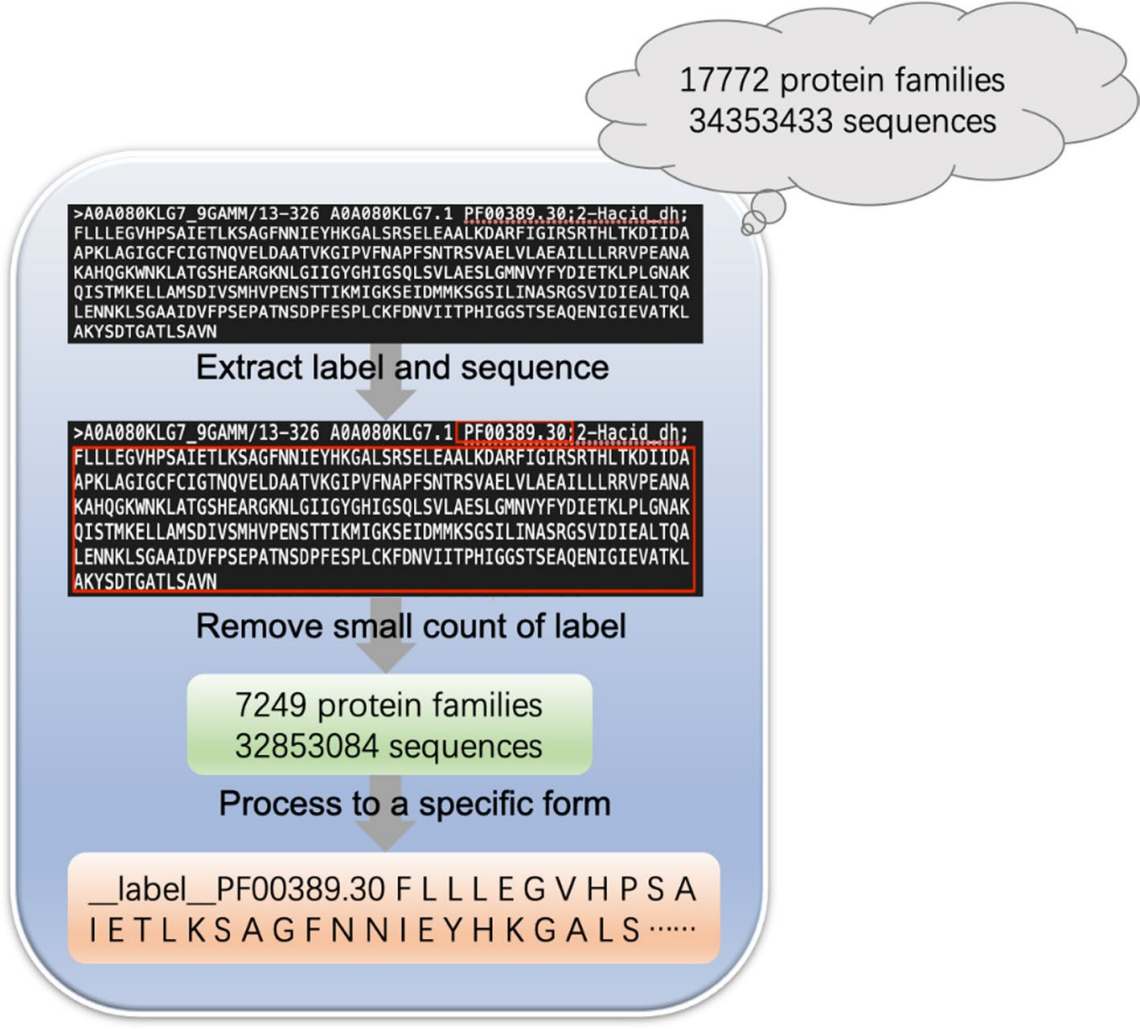
The data collection and filtering process of the Pfam database

We use a large-scale corpus, the Pfam database, to perform the supervised pre-training. The construction of Pfam database consists of the following steps (shown in Fig. 1):

(i) Download a total of 17,772 protein families and 34,353,433 sequences in the Pfam database of the version 32.0 released in 2019.
(ii) Extract label and sequence for each protein.
(iii) Construct a dataset including 7249 protein families and 32,853,084 sequences by deleting the protein families with less than 500 samples.

### ProtPlat model design

To describe the working principle of the ProtPlat model, we define the following notations.

$C$: The size of the feature space, i.e., the number of *k-*mers used for classification.

$m$: The dimension of the embedding representations.

$p$: The dimension of hidden layer.

$n$: The number of labels.

$V \in {\mathbb{R}}^{p*m}$: Input weight matrix.

$U \in {\mathbb{R}}^{n*p}$: Output weight matrix.

The workflow of the ProtPlat model can be formulated in the following.

Let $({x}^{(1)}, {x}^{(2)},\ldots,{x}^{(C)}) \epsilon {\mathbb{R}}^{m}$ be the input vectors. Pass them through a fully connected layer to get the embedding vectors,

3.1 $${h}^{(1)} = V \times {x}^{(1)},{h}^{(2)} = V \times {x}^{(2)},\ldots, {h}^{(C)} = V \times {x}^{(C)} \epsilon {\mathbb{R}}^{p}$$

Get the averaged embedding vector, $\widehat{h} \epsilon {\mathbb{R}}^{p}$

3.2 $$\widehat{h} = \frac{\sum i\in \{{1,2},\ldots,C\} {h}_{i}}{C}.$$

Then pass a fully connected layer to generate a score vector, $z \epsilon {\mathbb{R}}^{n}$

3.3 $$z = U \times \widehat{h}$$

Convert the score vector into the probability distribution of the label, $\widehat{y} \epsilon {\mathbb{R}}^{n}$

3.4 $$\widehat{y} = softmax(z)$$

**Fig. 2:**
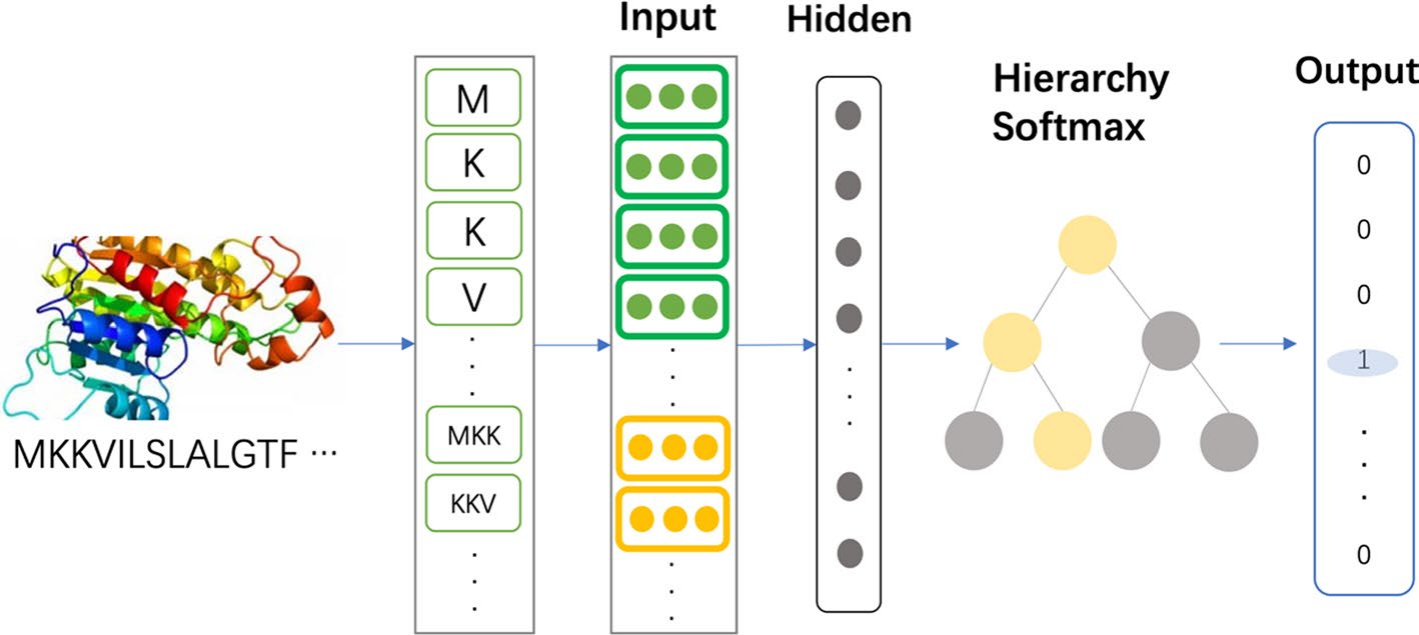
Model architecture of ProtPlat. The *k*-mer embeddings are fed into the neural network and learned by the hidden layers. The output label is yielded by a hierarchy Softmax function

The main structure of ProtPlat is a three-layer neural network, as shown in Fig. 2. The input is a $C\times m$-dimensional matrix, which consists of the vectors for words with length less than or equal to *k* in protein sequences. For instance, when *k* is set to 3, the input covers all amino acids, 2-mer and 3-mer features. The embedding vector of a *k*-mer is the average of the embedding vectors of the *k* amino acid vectors that it contains.

An important mechanism in the classification model is the hierarchical softmax function, which uses a binary tree to represent all categories. Each leaf node in the tree is a category, which can effectively ensure the efficient classification of a large number of labels. Hierarchical softmax is built based on Huffman coding [40], and the label is coded, which can greatly reduce the number of prediction targets of the model. The embedding representation of protein sequences in the model is a hidden variable, which can be reused. This architecture is similar to the CBow model [16], except that the central word in this model is replaced by a sequence label.

### The two-stage training procedure for downstream tasks

To apply ProtPlat to downstream tasks, we perform a two-stage training procedure.

(i) Pre-training

As described in Sect. 3.1, using the protein sequences and family labels in Pfam, we train the ProtPlat model. The input vectors for single amino acids are randomly initialized and the vector for a *k*-mer is the average of the *k* amino acid vectors that it contains. The output is the Pfam family labels. After training, we save the vector ${h}^{(i)}$ of each amino acid as its embedding representation, which is used as the input vector (i.e., ${x}^{(i)}$) for the downstream tasks.

(ii) Fine-tuning

The fine-tuning stage has almost the same training process as the pre-training. The differences lie in the input and output, where the input is pre-trained embedding vectors, and the output is the labels for the downstream classification task.

### Web server

**Fig. 3:**
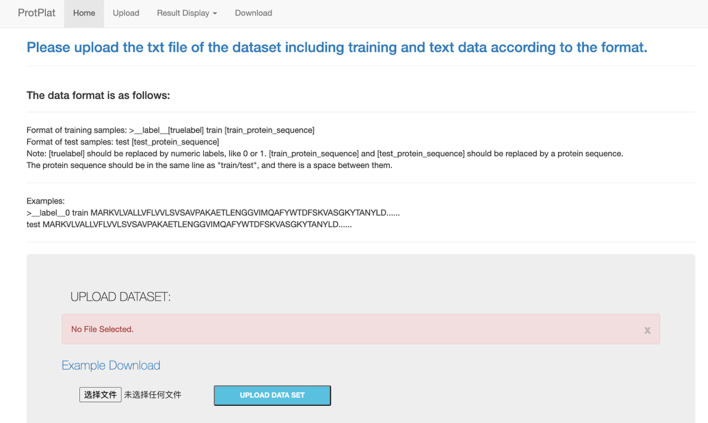
The web server interface of ProtPlat

We implement the ProtPlat platform as a web service (https://compbio.sjtu.edu.cn/protplat) that is accessible to the public. The web server interface is shown in Fig. 3. The background model of the web server has been pre-trained via the family classification task base on the Pfam database. Users can upload their own training and test sets to the server. The system will fine-tune the pre-trained model by using the uploaded training data and yield prediction results for the test data. After waiting for a while, the prediction results will be displayed on the web page. Besides, users can also download the embedding vectors of amino acids pre-trained by the platform (in the Download Tab).

In many protein classification problems, the training set is too small to support the learning of good representations from input data. Since many protein classification problems share common features extracted from their amino acid sequences, the small-data tasks can benefit a lot from the two-stage training strategy.

## Experimental results

### Experimental settings and evaluation metrics

For both the pre-training and fine-tuning, we randomly extract 20% of the training data to form the validation set. We select the best hyperparameters based on the model performance on the validation set. Table 5 shows the hyperparameter settings for the pre-training phase in ProtPlat. Note that the value of *k* is determined in the pre-training phase and remains unchanged in the downstream tasks. For each downstream task, the number of epochs and learning rate are tuned on its validation set.

**Table 5:** Hyperparameter settings for pre-training in ProtPlat

Hyperparameter	Value
*k*	3
Epoch	70
Learning rate	0.15
Dim. of embeddings	100
Dim. of hidden layer	100

To assess the performance of the model, we use four evaluation metrics in binary classification downstream tasks, including accuracy (ACC), F1 score, precision (Pre), and recall (Rec). They are formulated as follows.

4.1 $$ACC = \frac{TP + TN }{TP + TN + FP + FN},$$

4.2 $$F1 = \frac{2 * TP}{2 * TP + FP + FN},$$

4.3 $$Pre = \frac{TP}{TP + FP},$$

4.4 $$Rec = \frac{TP}{TP + FN},$$

where *TP*, *TN*, *FP*, and *FN* denote the numbers of true positive, true negative, false positive, and false negative, respectively. As for the multi-class problems, the F1 is defined as follows,

4.5 $$Pre= \frac{\sum {TP}_{i}}{\sum {TP}_{i}+\sum {FP}_{i}}, { where } \text{ i is the index of the category}$$

4.6 $$Rec= \frac{\sum {TP}_{i}}{\sum {TP}_{i}+\sum {F\text{N}}_{i}}, \text{where } \text{ i is the index of the category}$$

4.7 $$F1 = \frac{2 *Pre * Rec}{Pre + Rec},$$

where ${TP}_{i}$, ${FP}_{i}$, and ${F\mathrm{N}}_{i}$ denote the numbers of true positive, false positive, and false negative for the *i-*th class, respectively.

### Performance of the pre-training

**Fig. 4:**
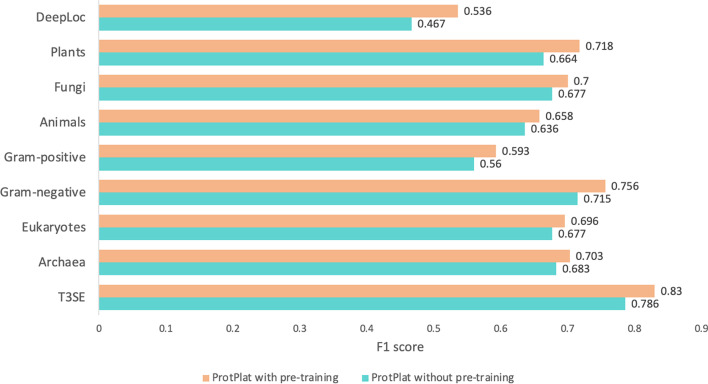
F1 score comparison between models with and without pre-training

Here we compare the models with and without pre-training. The model with pre-training uses pre-trained embedding vectors as the initial input, while the model without pre-training uses one-hot encoding vectors as the initial input and randomly initializes the input weights. We compare their performance on 9 datasets, including the T3SE dataset, three subcellular localization datasets (plants, fungi, and animals from BaCeILo [14] and the DeepLoc dataset [4]), and four signal peptide datasets (archaea, eukaryotes, Gram-positive, and Gram-negative). The results are shown in Fig. 4. As can be seen, pre-training can improve the prediction accuracy on all these datasets. The F1 value is increased by 0.03–0.08. Moreover, we perform a statistical significance analysis on the performance difference. For each dataset of the downstream tasks, we run the models with and without pre-training for 30 times, respectively. The p-values of the pair-wise *t*-test are listed in Additional file 1: Table S2 and S4. For all the 8 downstream tasks, the p-values are much less than 0.01, indicating that the pre-trained model is significantly superior to the model without pre-training.

### Comparison of ProtPlat with the existing methods on three downstream tasks

#### Task I: identification of type III secreted effectors

Identification of type III secreted effectors is a binary classification problem, i.e., T3SE and non-T3SE. To evaluate the performance of ProtPlat, we compare it with the existing representative methods, including WEDeepT3 [13], BPBAac [17], EffectiveT3 [18], T3_MM [19], DeepT3 [20], Bastion3 [21] and BEAN 2.0 [22]. The results of ProtPlat obtained on the test set of WEDeepT3 are shown in Table 6. As can be seen, ProtPlat has achieved the best performance. Compared with the second-best model WEDeepT3, the F1 value of the pre-trained ProtPlat model has increased by 0.128, and the total accuracy has increased by 0.021, which confirms the classification performance of the pre-training platform. Since F1 score is threshold-dependent, we compute the F-max metric (shown in Additional file 1: Table S6). The F1 scores under different thresholds are shown in Additional file 1: Figure S1.

**Table 6:** Performance comparison for the prediction of type III secreted effectors

Model	ACC	F1 score
ProtPlat	**0.833**	**0.833**
WEDeepT3	0.812	0.705
BPBAac	0.609	0.339
EffectiveT3	0.696	0.512
T3_MM	0.718	0.581
DeepT3	0.594	0.486
Bastion3	0.739	0.673
BEAN 2.0	0.761	0.692

The accuracy and F1 scores of the baseline methods are extracted from [13]. All the methods are evaluated on the same test set.

#### Task II: prediction of protein subcellular location

For protein subcellular localization, we use the dataset in BaCeILo [14] and compare with Euk-mPLoc [23], LOCTree [24], BaCeILo [14], and YLoc [25]. The prediction performance is evaluated by accuracy and F1 score. The F-max metric and F1 scores under different thresholds are shown in Additional file 1: Table S6 and Figure S1. For all the three datasets (plants, fungi, and animals), ProtPlat achieves competitive performance. Especially on the Fungi dataset, ProtPlat outperforms other models by a large margin (both F1 and accuracy are increased by over 10%) (Table 7), indicating that small datasets may benefit more from the pre-training. Note that the training sets of the baseline models are different [25], and many of the baseline models are more general predictors, which can predict more than 4 locations, like YLoc-HighRes, YLoc + , MultiLoc2-HighRes, WoLF PSORT, Euk-mPLoc, and LOCTree. Thus, they may perform worse than the predictors specifically trained for these four locations. The accuracy and F1 scores of the baseline methods are extracted from YLoc [25].

**Table 7:** Performance comparison for protein subcellular location prediction

Model	Animals		Fungi		Plants	
	ACC	F1	ACC	F1	ACC	F1
Euk-mPLoc	0.61	0.54	0.6	0.56	0.46	0.37
WoLF PSORT	0.7	0.67	0.5	0.51	0.57	0.46
LOCTree	0.62	0.58	0.47	0.43	0.7	0.58
BaCeILo	0.64	0.66	0.57	0.6	0.69	0.56
MultiLoc2-HighRes	0.68	0.71	0.53	0.58	0.62	0.54
MultiLoc2-LowRes	0.73	**0.76**	0.6	0.61	**0.76**	0.64
YLoc +	0.58	0.67	0.48	0.51	0.58	0.49
YLoc-HighRes	0.74	0.69	0.56	0.51	0.58	0.54
YLoc-LowRes	**0.79**	0.75	0.56	0.61	0.71	0.58
ProtPlat	0.66	0.66	**0.71**	**0.71**	0.72	**0.72**

It is worth noting that almost all the baseline methods utilize information from multiple sources as input features, including some kinds of domain knowledge, such as protein functional domain and Gene Ontology. By contrast, ProtPlat uses sequence information and the protein family labels in Pfam, which are general information for proteins and not specific to prediction tasks, while it can obtain comparable or even better results than the baseline methods.

The comparison results suggest the powerful learning ability of the pre-training platform, which would be very useful when domain knowledge is scarce.

We also compare ProtPlat with the state-of-the-art methods on the DeepLoc dataset. The accuracy of ProtPlat is much lower than DeepLoc (results shown in Additional file 1: Table S3). The reason is mainly due to the imbalanced distribution of the DeepLoc dataset. As described in Sect. 2 (Task II), there are 10 classes in this dataset, and the largest class has 4043 samples while the smallest one has only 154 samples (shown in Additional file 1: Table S1). The DeepLoc adopts a cost matrix-based method to mitigate the effect of class imbalance, while there is no specific operation in our model for dealing with this issue. The other two methods, LocTree2 [44] and YLoc [25], also perform better than ProtPlat, as both utilize some domain knowledge. Besides the sequence features, i.e., amino acid composition and pseudo composition, YLoc also uses the PROSITE motifs and GO terms from the homologs of the query protein. LocTree2’s input includes sequences as well as their profiles searched via PSI-BLAST [45], and it incorporates similarities among subcellular locations in the design of SVM classifiers.

#### Task III: signal peptide prediction

For the recognition of signal peptides, we perform binary classification and compare ProtPlat with 16 baseline methods mentioned in SignalP 5.0 [15]. The prediction performance is evaluated by precision, recall, and F1. Results are shown in Table 8. The F-max metric and F1 scores under different thresholds are shown in Additional file 1: Table S6 and Figure S1. For the Archaea and Gram-negative datasets, ProtPlat has the highest F1 scores. In general, the performance of ProtPlat is comparable to SignalP 5.0, which uses hand-crafted features and specific architecture for recognizing signals, and higher than all the other 15 baselines. For Eukaryotes dataset, the precision and recall values of ProtPlat are closer to SignalP 5.0 and higher than those of other baselines. For Gram-negative dataset, the result of ProtPlat is close to that of SignalP 5.0, where the precision value is significantly higher than other baselines and the recall value is also higher. For the Gram-positive dataset, ProtPlat achieves comprehensively better performance, and the precision is significantly higher than other baselines.

**Table 8:** Performance comparison of signal peptide prediction

Model	Archaea			Eukaryotes			Gram-negative			Gram-positive		
	Pre	Rec	F1	Pre	Rec	F1	Pre	Rec	F1	Pre	Rec	F1
SignalP 5.0	0.771	0.660	0.711	**0.671**	0.729	**0.699**	**0.742**	0.733	0.737	**0.600**	**0.840**	**0.700**
DeepSig	–	–	–	0.604	0.624	0.614	0.131	0.600	0.215	0.073	0.760	0.133
LipoP	0.484	0.480	0.482	0.159	0.343	0.217	0.327	0.733	0.452	0.153	0.600	0.244
Philius	0.425	0.580	0.491	0.151	0.619	0.243	0.106	0.700	0.184	0.054	0.600	0.099
Phobius	0.395	0.540	0.456	0.226	0.667	0.338	0.098	0.644	0.170	0.054	0.600	0.099
PolyPhobius	0.395	0.560	0.463	0.176	0.681	0.280	0.097	0.644	0.169	0.060	0.680	0.110
PrediSi	–	–	–	0.273	0.652	0.385	0.144	0.722	0.240	0.062	0.640	0.113
PRED-LIPO	0.455	0.480	0.467	0.069	0.095	0.080	0.212	0.467	0.292	0.216	0.760	0.336
PRED-SIGNAL	0.489	**0.800**	0.607	0.066	0.224	0.102	0.076	0.444	0.130	0.060	0.680	0.110
PRED-TAT	0.493	0.580	0.533	0.080	0.410	0.134	0.125	0.711	0.213	0.082	0.720	0.147
Signal-3L 2.0	–	–	–	0.322	0.648	0.430	0.113	0.644	0.192	0.074	0.800	0.135
Signal-CF	–	–	–	0.105	0.652	0.181	0.102	0.689	0.178	0.059	0.720	0.109
SOSUIsignal	–	–	–	0.037	0.176	0.061	0.040	0.267	0.070	0.018	0.200	0.033
SPEPlip	–	–	–	0.366	0.710	0.483	0.276	0.611	0.380	0.187	0.680	0.293
SPOCTOPUS	–	–	–	0.120	0.390	0.184	0.067	0.467	0.117	0.056	0.640	0.103
TOPCONS2	0.366	0.480	0.415	0.107	0.371	0.166	0.081	0.544	0.141	0.022	0.240	0.040
**ProtPlat**	**0.823**	0.627	**0.712**	0.636	**0.773**	0.698	0.728	**0.791**	**0.758**	0.550	0.668	0.603

*Pre denotes precision and Rec denotes recall. The precision and recall of the baseline methods are extracted from SignalP 5.0 [15]

### Ablation studies on ProtPlat

#### Comparison of the two segmentation methods

We experiment with both the non-overlapping and overlapping segmentation methods. The results on the three downstream tasks are shown in Table 9. The model settings are the same. Different segmentation strategies lead to different input vectors. The results show that when using the overlapping segmentation, the embedding vectors pre-trained by the model lead to a better F1 score for all the tasks.

**Table 9:** Comparison of the F1 Scores between two segmentation methods

Dataset	Non-overlapping segmentation	Overlapping segmentation
T3SE	0.792	**0.833**
Animals	0.623	**0.660**
Fungi	0.688	**0.709**
Plants	0.671	**0.723**
Archaea	0.679	**0.712**
Eukaryotes	0.680	**0.698**
Gram-negative	0.713	**0.758**
Gram-positive	0.558	**0.603**

#### Investigation on the value of *k*

**Fig. 5:**
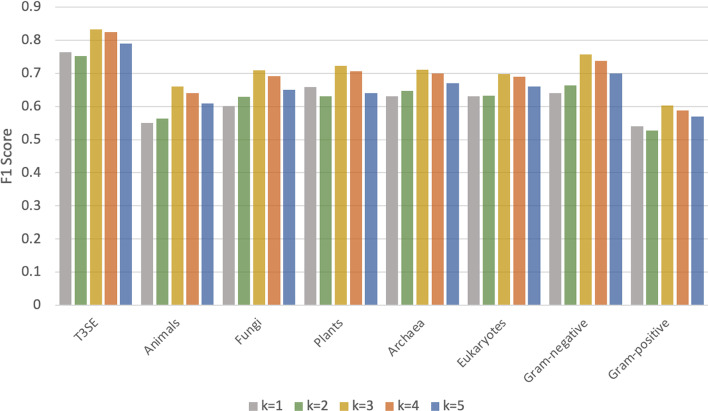
Comparison of F1 scores obtained by ProtPlat with different values of *k*

In ProtPlat, the value of *k* is set to 3, which is determined in the pre-training phase, i.e., based on the performance on the protein family classification task. To investigate whether it is a good choice for the downstream tasks, here we assess the model performance under different values of *k*. Figure 5 shows the F1 scores for the downstream tasks when *k* is set to 1, 2, 3, 4, and 5, respectively, using the overlapping segmentation for protein sequences. The results show that the best performance is achieved when *k* is set to 3 for all the tasks, suggesting that the protein sequence-based classification tasks share sequence features and the pre-training can transfer knowledge to other tasks. When *k* is set to 1, each amino acid is treated independently and contextual information (i.e., local sequence information) is not included, thus the performance is not good. When *k* is equal to or greater than 5, the accuracy drops because the *k*-mer space has extremely high dimensionality, containing a lot of rare *k*-mers (with a very low frequency), which may lead to the overfitting issue.

### Comparison with other pre-trained protein representations

We compare ProtPlat with two state-of-the-art protein pre-training models, i.e., SeqVec [26] and ProtTrans [27], on all the downstream task datasets. The results are shown in Table 10. Besides, we perform a statistical significance analysis for the performance different of three pre-training methods shown in Additional file 1: Table S5. We use the 1024-dimensional pre-trained embedding vectors taken from SeqVec and the ProtAlbert model in ProtTrans as the input of ProtPlat.

**Table 10:** Accuracy of different pre-trained representations

Dataset	Training No.	ProtPlat	SeqVec	ProtTrans
DeepLoc	11,085	0.537	0.565	**0.582**
T3SE	525	**0.836**	0.823	0.821
Animals	1890	0.665	0.685	**0.694**
Fungi	1010	0.706	0.727	**0.742**
Plants	204	0.718	**0.741**	0.738
Archaea	55	**0.729**	0.718	0.714
Eukaryotes	9813	0.695	0.721	**0.738**
Gram-negative	1545	0.755	0.772	**0.782**
Gram-positive	534	0.607	0.614	**0.628**

As can be seen, ProtPlat achieves the best performance on two relatively small datasets, especially the Archaea dataset, which contains only 55 training samples. The reason is that the embedding vectors yielded by SeqVec and ProtTrans have a high dimensionality (1024), which may result in the overfitting issue, while our model only uses 100-dimensional embedding vectors.

Although ProtPlat seems to have little advantage in the comparison with SeqVec and ProtTrans regarding the prediction accuracy, ProtPlat is a lightweight, cost-effective, and highly efficient model. Different from SeqVec and ProtTrans, both of which are based on language modeling to perform large-scale unsupervised pretraining, ProtPlat adopts supervised learning, i.e., the protein family classification in Pfam, as the pretraining task. For ELMo-based SeqVec, it was trained for three weeks on 5 Nvidia Titan GPUs with 12 GB memory each. As mentioned in Background, ProtTrans uses various transformer models, which were trained on a supercomputer with 936 nodes (total 5616 GPUs) and one TPU Pod (V3-512 or V3-1024). By contrast, ProtPlat takes only several CPU hours for pre-training. Therefore, it could also serve as an alternative pre-training model for protein-related prediction tasks, especially when the downstream task has very limited training samples.

## Discussion

This study proposes a pre-training platform to address the contradiction between a large number of protein sequences and the small scale of training data for various protein classification problems. The advantages of ProtPlat are mainly two folds.

(i) Fast and lightweight. ProtPlat does not use evolutionary information in the process of training, but only uses sequence information for prediction. It is a simple model with a few training parameters and fast training speed. We can train ProtPlat for classifying half a million sentences among 312 K classes in less than a minute using a standard CPU without GPU support.
(ii) Suitable for small datasets. ProtPlat model has especially good performance on small data sets. Since small data is usually insufficient for training, the pre-training procedure that provides a good initial model has a more obvious effect. Our experimental results also show that the classification of small datasets benefits more from the platform.

A limitation of this study is that we only consider the protein-level classification tasks, while a lot of prediction tasks are at the residue-level, such as secondary structure prediction and residue contact map prediction. Although the word embeddings learned in the system represent single amino acids or *k*-mers, the residue-level prediction has not been supported in the current version yet. One of our future works is to incorporate the residue-level prediction function into our platform and make it more general for protein-related computation tasks.

## Supplementary Information

**Additional file 1. Table S1:** Information of DeepLoc dataset, **Table S2:** Significance analysis of accuracy for models with (w) and without (w/o) pre-training, **Table S3:** Performance comparison on the DeepLoc dataset, **Table S4:** Significance analysis of F1 score for models with (w) and without (w/o) pre-training, **Table S5:** Significance analysis of accuracy for different pre-trained representations, **Table S6:** F-max on downstream tasks, **Figure S1:** F1 scores under different thresholds for 8 downstream task datasets.

## Data Availability

We adopt the same dataset as WEDeepT3 [13] for the type III secreted effectors prediction. For subcellular location prediction, we use a classic benchmark set, BaCeILo [14], including proteins from animals, fungi, and plants. We adopt the same dataset as SignalP 5.0 [15] for signal peptide prediction. The datasets used and analyzed during the current study available from the corresponding author on reasonable request. Data availability: For the three downstream tasks, we use open-source datasets of other models.
