# Supplementary material for: ProtPlat: an efficient pre-training platform for protein classification based on FastText
Source: BMC Bioinformatics. 2022 Feb 11;23:66. doi: 10.1186/s12859-022-04604-2 (PMC8832758; doi:10.1186/s12859-022-04604-2)
Supplement: Supplementary file 1 — Additional file 1. Table S1: Information of DeepLoc dataset, Table S2: Significance analysis of accuracy for models with (w) and without (w/o) pre-training, Table S3: Performance comparison on the DeepLoc dataset, Table S4: Significance analysis of F1 score for models with (w) and without (w/o) pre-training, Table S5: Significance analysis of accuracy for different pre-trained representations, Table S6: F-max on downstream tasks, Figure S1: F1 scores under different thresholds for 8 downstream task datasets. [file 12859_2022_4604_MOESM1_ESM.pdf]

Supplementary Materials

1 Tables

Table S1 Information of DeepLoc dataset

Location	# protein sequences
Nucleus	4043
Cytoplasm	2542
Extracellular	1973
Mitochondrion	1510
membrane	1340
Endoplasmic	862
Plastid	757
Golgi	356
Lysosome	321
Peroxisome	154

Table S2 Significance analysis of accuracy for models with (w) and without (w/o) pre-training

Dataset	Mean value		Variance		p-value
	w/o pre	w pre	w/o pre	w pre	
T3SE	0.786	0.830	4.29E-05	3.34E-05	6.39E-12
Animals	0.636	0.658	0.05E-05	1.68E-05	1.07E-08
Fungi	0.677	0.700	3.41E-05	6.37E-05	9.53E-07
Plants	0.664	0.718	3.79E-05	5.47E-05	8.25E-13
Archaea	0.765	0.790	5.13E-05	3.24E-05	3.65E-07
Eukaryotes	0.923	0.947	3.21E-05	1.58E-05	9.13E-09
Gram-negative	0.715	0.751	4.97E-05	8.62E-06	3.69E-10
Gram-positive	0.784	0.810	3.47E-05	6.53E-05	3.68E-09

Table S3 Performance comparison on the DeepLoc dataset

Models	Accuracy
DeepLoc	0.780
LocTree2	0.612
YLoc	0.612
ProtPlat	0.536

Table S4 Significance analysis of F1 score for models with (w) and without (w/o) pre-training

Dataset	Mean value		Variance		p-value
	w/o pre	w pre	w/o pre	w pre	
T3SE	0.786	0.830	4.29E-05	3.34E-05	6.39E-12
Animals	0.636	0.658	0.05E-05	1.68E-05	1.07E-08
Fungi	0.677	0.700	3.41E-05	6.37E-05	9.53E-07
Plants	0.664	0.718	3.79E-05	5.47E-05	8.25E-13
Archaea	0.683	0.703	8.47E-05	4.27E-05	2.56E-05
Eukaryotes	0.677	0.696	1.71E-05	2.01E-05	1.68E-08
Gram-negative	0.715	0.756	1.39E-05	2.42E-05	4.50E-14
Gram-positive	0.56	0.593	4.31E-05	5.32E-05	3.61E-09

Table S5 Significance analysis of accuracy for different pre-trained representations

Dataset	Mean value			Variance			p-value
	ProtPlat	SeqVec	ProtTrans	ProtPlat	SeqVec	ProtTrans	
DeepLoc	0.537	0.565	0.582	7E-06	4.3E-06	5.8E-06	5.59E-12
T3SE	0.836	0.823	0.821	4.67E-04	7.50E-06	2.17E-06	0.046
Animals	0.665	0.685	0.694	1.2E-04	7.7E-06	9.3E-06	4.90E-05
Fungi	0.706	0.727	0.742	3.18E-05	2.15E-05	4.31E-05	8.17E-08
Plants	0.718	0.741	0.738	1.22E-05	3.8E-06	3.8E-06	1.25E-08
Archaea	0.729	0.718	0.714	6.3E-06	4.5E-06	1.7E-06	2.46E-07
Eukaryotes	0.695	0.721	0.738	2.5E-06	3.8E-06	6.5E-06	1.61E-12
Gram-negative	0.755	0.772	0.782	1.57E-05	4E-06	4E-06	1.41E-08
Gram-positive	0.607	0.614	0.628	4.7E-06	2.5E-06	4.3E-06	4.68E-09

Table S6 F-max on downstream tasks

Dataset	F-max
T3SE	0.848
Animals	0.66
Fungi	0.715
Plants	0.757
Archaea	0.72
Eukarya	0.698
Gram-negative	0.767
Gram-positive	0.654

2 Figures

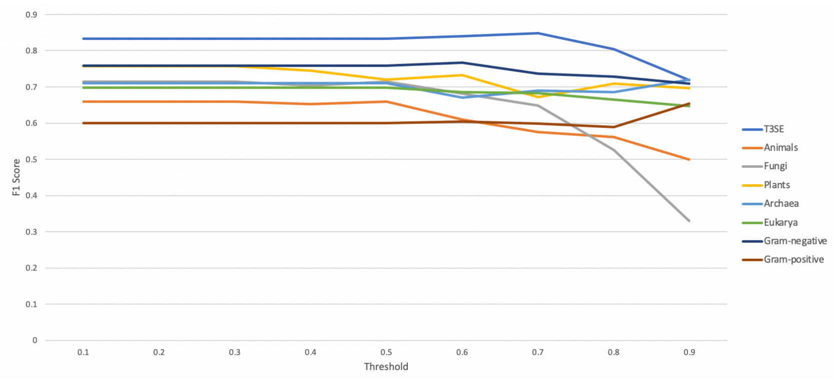

Figure S1 F_1 scores under different thresholds for 8 downstream task datasets
